# Management of Hyponatremia in Heart Failure: Practical Considerations

**DOI:** 10.3390/jpm13010140

**Published:** 2023-01-10

**Authors:** Victoriţa Şorodoc, Andreea Asaftei, Gabriela Puha, Alexandr Ceasovschih, Cătălina Lionte, Oana Sîrbu, Cristina Bologa, Raluca Ecaterina Haliga, Mihai Constantin, Adorata Elena Coman, Ovidiu Rusalim Petriș, Alexandra Stoica, Laurenţiu Şorodoc

**Affiliations:** 1 2nd Internal Medicine Department, Sf. Spiridon Clinical Emergency Hospital, 700111 Iasi, Romania; 2 2nd Rheumatology Department, Clinical Rehabilitation Hospital, 700661 Iasi, Romania; 3 Internal Medicine Department, Faculty of Medicine, Grigore T. Popa University of Medicine and Pharmacy, 700115 Iasi, Romania

**Keywords:** heart failure, hyponatremia, dilutional, arginine vasopressin, loop diuretics, osmotic demyelination syndrome, hypertonic saline, vaptans, conivaptan, tolvaptan

## Abstract

Hyponatremia is commonly encountered in the setting of heart failure, especially in decompensated, fluid-overloaded patients. The pathophysiology of hyponatremia in patients with heart failure is complex, including numerous mechanisms: increased activity of the sympathetic nervous system and the renin–angiotensin–aldosterone system, high levels of arginine vasopressin and diuretic use. Symptoms are usually mild but hyponatremic encephalopathy can occur if there is an acute decrease in serum sodium levels. It is crucial to differentiate between dilutional hyponatremia, where free water excretion should be promoted, and depletional hyponatremia, where administration of saline is needed. An inappropriate correction of hyponatremia may lead to osmotic demyelination syndrome which can cause severe neurological symptoms. Treatment options for hyponatremia in heart failure, such as water restriction or the use of hypertonic saline with loop diuretics, have limited efficacy. The aim of this review is to summarize the principal mechanisms involved in the occurrence of hyponatremia, to present the main guidelines for the treatment of hyponatremia, and to collect and analyze data from studies which target new treatment options, such as vaptans.

## 1. Introduction

Hyponatremia, defined as serum sodium level below 135 mEq/L, is the most common electrolyte disorder in patients with heart failure [1]. For one in five patients, hyponatremia is evident on admission and has approximately the same extent throughout hospitalization [2,3,4]. Many studies demonstrate the strong prognostic value of hyponatremia being associated with a prolonged hospital length of stay, higher risk of readmission, and in hospital and after discharge mortality [5,6]. There is no clear indication yet whether hyponatremia in itself influences the prognosis or whether it just occurs more often in patients with heart failure. In addition, it is equally unclear whether it is a repercussion of diuretic therapy, which leads to patients being discharged with congestion, which, in turn, is associated with decreased survival. In the majority of cases the mechanism of hyponatremia is dilutional hypervolemic and it should be differentiated from the depletional process because these two conditions need a different approach [7]. 

## 2. Dilutional Hyponatremia

Heart failure related hyponatremia is thought to have a multidimensional pathogenesis. It usually occurs in patients with advanced chronic heart failure when ventricular dysfunction is severe. It results from a synergistic interaction between increased secretion of arginine vasopressin (AVP), enhanced activity of the sympathetic nervous system, and the renin–angiotensin–aldosterone system (RAAS) [7]. 

The reduced cardiac output of patients with advanced heart failure leads to the activation of the sympathetic nervous system. By inducing peripheral vasoconstriction, coupled with reduced cardiac output, this phenomenon results in water and sodium retention and RAAS activation, leading to an increase in angiotensin II levels. Angiotensin II can cause a worsening of hyponatremia in multiple ways: inducing water and sodium retention by numerous renal mechanisms, thirst stimulation, and the release of AVP. High levels of AVP are also caused by the decreased effective arterial filling which leads to a breakdown of baroreceptor-mediated inhibition of AVP release [8]. 

AVP, also called the antidiuretic hormone (ADH), plays a major role in heart failure associated hyponatremia. It is synthesized in the paraventricular and supraoptic nuclei of the anterior hypothalamus and stored in the posterior lobe of the pituitary gland [9]. AVP is released into the bloodstream in response to osmotic stimuli (plasma hypertonicity) or non-osmotic stimuli (decreased cardiac output, intravascular volume, or blood pressure). Taking into consideration that plasma osmolality in heart failure is normal or low, the predominance of non-osmotic AVP secretion over osmotic AVP secretion plays the main role in the development of hyponatremia [10]. Investigators in the SOLVD (Studies of Left Ventricular Dysfunction) trial concluded that in patients with ejection fractions of 35% or less, the level of AVP is significantly higher than in healthy patients [11]. 

**Figure 1:**
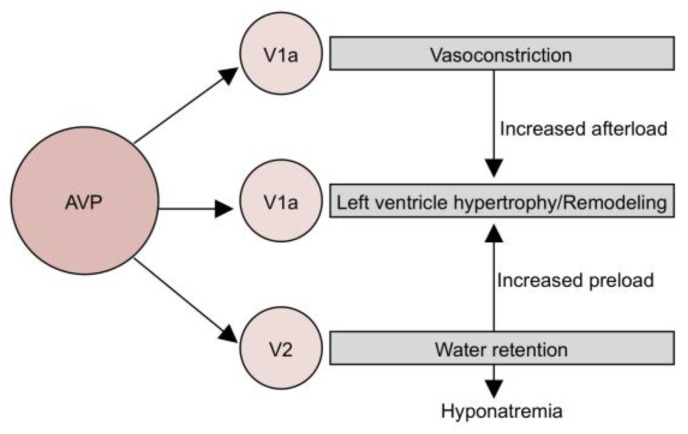
Effects of AVP in heart failure—the vicious cycle.

By binding to V2R in the collecting ducts of the nephron, AVP increases the aquaporin-2 water channel expression. As a result, AVP increases water permeability and enhances free water retention [12]. By binding to V1aR, found in the walls of arteries, arterioles, and veins, AVP leads to vasoconstriction, causing an increased vascular resistance and afterload. In case of chronic stimulation of V1aR and high levels of AVP, coronary vasoconstriction appears, leading to a decrease in coronary blood flow and cardiac contractility [13]. A study conducted by Hiroyama M. et al., where rat cardiac fibroblasts were stimulated with AVP, concluded that V1aR activation promotes cardiomyocyte hypertrophy by stimulating cardiac fibroblast proliferation. In the end, excessive AVP-V1aR signaling leads to changes in cardiac morphology and contractility [14]. The negative effects of excessive stimulation of both V1aR and V2R receptors are summarized in Figure 1. 

## 3. Depletional Hyponatremia

Depletional hyponatremia can occur as a consequence of gastro-intestinal and third-space losses, osmotic diuresis induced by hyperglycemia in severe uncontrolled diabetes, salt-restricted diets followed by patients with heart failure, and administration of diuretic agents. Diuretic therapy represents the most frequent cause of depletional hyponatremia [7].

When managing heart failure patients, diuretics represent a key part of the treatment, taking into consideration that higher rates of rehospitalization and mortality are associated with congestion persistent at discharge [2].

Intravenous loop diuretics are the background therapy in acute decompensated heart failure (ADHF), and the majority of patients will require prescription of loop diuretics at discharge in order to lower the risk of symptom recurrence and rehospitalization. Achieving an effective diuresis may necessitate doubling the initial dose of diuretic, adding a thiazide diuretic, or adding a mineralocorticoid receptor antagonist which also has cardiovascular protection properties [15]. 

The adverse effects associated with diuretic therapy usually depend on the type and dosage of the diuretic. Dyselectrolitemia (hyponatremia, hypovolemia, hypokalemia) and fluid balance abnormalities are the most commonly encountered adverse effects [16].

Loop diuretics act in the thick ascending limb of the loop of Henle, inhibiting sodium chloride (NaCl) reabsorption, blocking the initial step in the development of a hyperosmotic gradient in the medullary interstitium [17]. Normally, the highly concentrated interstitium, in the presence of vasopressin, allows water reabsorption in the medullary collecting tubule and induces the excretion of a concentrated urine. A loop diuretic interferes with this mechanism by compromising the accumulation of NaCl in the medulla. Although volume depletion induced by a loop diuretic can increase vasopressin levels (leading to dilutional hyponatremia), the impairment in the medullary gradient reduces the responsiveness to AVP. This results in limited water retention and a reduced risk of developing hyponatremia, as long as the water intake is not very high, or the distal delivery is not very low [7].

An important problem which occurs during the treatment of patients with heart failure is represented by diuretic resistance (DR). In this group of patients, the prevalence of DR is estimated as 20–30% [18]. It is associated with a higher risk of subsequent death, rehospitalization, or renal complications from congestive heart failure [19]. Loop diuretics have complex effects on renal and systemic hemodynamics, which are influenced by the dose, route, and chronicity of administration. Braking phenomenon, rebound sodium retention, and renal adaptation are the main mechanisms involved in DR. Studies in rats proved that chronic use of loop diuretics leads to hypertrophy and hyperplasia in epithelial cells of the distal convoluted tubule—known as the braking phenomenon [20]. Chronic diuretic therapy further intensifies this nephron remodeling by increasing the levels of angiotensin II and aldosterone (due to sodium and water loss). Consequently, there is an increase in sodium reabsorption, diminishing the natriuretic response [18].

In order to avoid the risk of developing diuretic resistance, a thiazide diuretic can be associated with loop diuretic therapy—the so-called sequential nephron blockade. In this way, increased sodium reabsorption due to renal adaptation can be avoided [21]. In comparison to loop diuretics, thiazide diuretics reduce the reabsorption of NaCl in the distal renal tubules (which is the main diluting site of the nephron) by blocking the thiazide-sensitive Na^+^/Cl^−^ cotransporter [22]. 

Diuretic induced hyponatremia is more likely to be induced by a thiazide-type diuretic than a loop diuretic due to differences in their tubular site of action. Depletional hyponatremia can be managed with saline administration and with the discontinuation of distally working diuretics (mineralocorticoid receptor antagonists, amiloride, thiazide-type) [7].

## 4. Clinical Aspects in Hyponatremia

Hyponatremia can produce a variety of symptoms depending on its degree and chronicity. In case of a gradual decrease in sodium levels (greater than 48 h) or mild hyponatremia, patients are usually asymptomatic/pauci-symptomatic [23]. Symptoms such as fatigue, nausea, vomiting, dizziness, ataxia, headache, confusion, and muscle cramps can be present in the case of a mild/moderate hyponatremia [24].

When a significant decrease in serum Na occurs acutely, the symptoms are more severe, and they define hyponatremic encephalopathy. Brain edema is a consequence of the brain losing the ability to regulate its volume by electrolyte losses when hypo-osmolality arises at a high level. Hyponatremic encephalopathy is associated with a mortality rate of 34% [25].

In chronic hyponatremia brain swelling is counteracted by the presence of adaptive mechanisms. As a first adaptive response, the interstitial fluid is displaced into the cerebrospinal fluid and then into the bloodstream. The second mechanism, and also the more sustained one, is the ‘volume regulatory decrease’ (VRD) which involves the extrusion of osmotically active ions in order to reduce cellular swelling [26]. Glutamate is one of the lost organic osmolytes and due to its neuroactive properties and involvement in synaptic release of excitatory neurotransmitters, neurological abnormalities observed in chronically hyponatremic patients can be explained [27].

**Figure 2:**
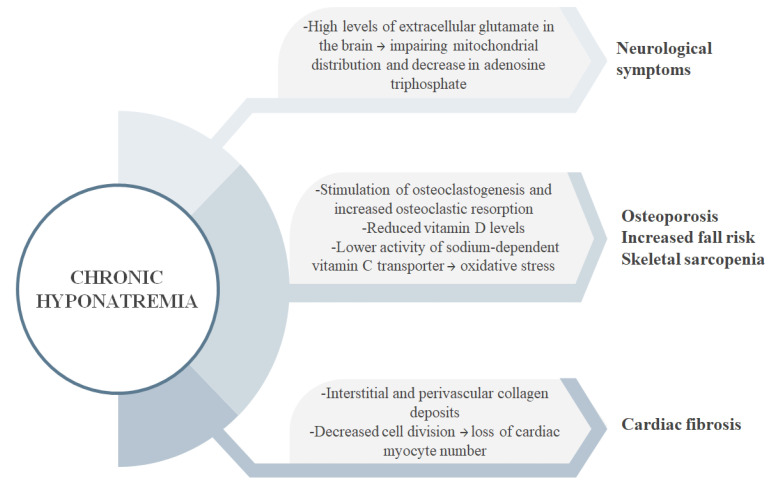
Effects of chronic hyponatremia.

Even though hyponatremia was considered to be asymptomatic due to adaptive mechanisms in the brain, recent studies proved that chronic hyponatremia may be associated with cognitive impairments, gait disturbances, low bone mineral density, and a higher risk of falls [28,29]. These impairments (Figure 2) affect the quality of life and may represent a significant cause of mortality in people with heart failure and chronic hyponatremia.

In the context of a low serum sodium concentration which causes gait impairment and a higher risk of osteoporosis by stimulating the osteoclastic activity and bone resorption, patients are predisposed to falls and fractures, which may raise the rehospitalization and mortality rates [30]. Barsony et al., also demonstrated that in chronic hyponatremia there is a lower activity of sodium-dependent vitamin C transporter. It is well-known that ascorbic acid is involved in maintaining the equilibrium between osteoblastic and osteoclastic processes, as well as in protection against oxidative stress. In consequence, a lower uptake of vitamin C leads to production of free reactive oxygen species, increasing the degenerative processes in the organism [31]. In a study done on aged rats in which hyponatremia was induced for a period of 18 weeks, results showed that chronic low levels of serum sodium are also associated with changes in the morphology of the heart. Heart weights were increased in hyponatremic rats compared to normonametric rats, a process explained by cardiac fibrosis caused by interstitial and perivascular collagen deposits. A loss of cardiac myocyte number was also found in hyponatremic rats, due to an impairment in cell division [32].

By inducing hyponatremia in rats with continuous injections of 1-deamino-8-D-arginine vasopressin (dDAVP) (a V2 receptor agonist), Fujisawa H. et al., showed the association between low sodium serum levels and memory impairment, attention deficits, and gait disturbances [28]. As mentioned before, the neurological abnormalities are caused by the low intracellular glutamate levels in the brain. Sodium-dependent glial glutamate transporters, GLT-1 and GLAST, play the main role in the astrocytic glutamate uptake mechanism and in maintaining a low extracellular glutamate concentration [33]. A study done by Verbalis et al., concluded that sustained low sodium serum levels in rats are associated with a 38.6% decrease in the brain content of glutamate after 14 days [34]. The excessive levels of the extracellular glutamate may have a toxic effect on the brain, resulting in an impairing mitochondrial distribution and a decrease in the ATP (adenosine triphosphate) content of neurons. This suggests the direct effect of hyponatremia in inducing neuronal dysfunction [35]. Studies showed that these neurological and behavioral abnormalities are reversible. By correcting chronic hyponatremia (which was maintained for 3–4 weeks) with tolvaptan in rats, gait performances, memory, and recognition were equivalent to those in the control rats. [28].

An inappropriate correction of hyponatremia may lead to osmotic demyelination syndrome (ODS). It represents a non-inflammatory demyelination in the central pons, thalami, hippocampi, basal ganglia, and peripheral cortex which appears in response to an osmotic challenge [36]. Pathophysiologically, there is a rapid water movement out of the brain cells with an acute decrease in brain cell volume [37]. The diagnosis is usually clinical, based on the neurological symptoms (Table 1) which occur after a rapid correction of sodium levels (usually after 2–6 days). For confirmation, MRI imaging can be used to identify the typical lesions [38].

There are several factors which determine the risk of developing ODS. Patients with chronic hyponatremia (especially if starting serum sodium is <120 mmol/L), concomitant hypokalemia, alcoholism, malnutrition, and advanced liver diseases are at higher risk [39]. There is a lack of effective and validated therapies for ODS, while preventing ODS occurrence by not exceeding the recommended limits of hyponatremia correction is the main goal.

The daily limit of serum sodium correction recommended by the European Clinical Practice Guideline is 10 mEq/L in the first 24 h and 8 mEq/L during every 24 h thereafter [40]. In a literature review done by Tandukar et al., it was concluded that despite this recommendation, ODS can occur even in the case of adherence to current hyponatremia correction guidelines. They recommend a serum sodium correction of <8 mEq/L in patients with severe hyponatremia [36].

## 5. Diagnosis of Heart Failure Associated Hyponatremia

The first step in approaching hyponatremia is to determine the plasma tonicity in order to exclude other factors that can be associated with hyponatremia. This can be done by measuring plasma osmolality. In the case of isotonic hyponatremia (plasma osmolality: 285–295 mOsm/L) serum sodium levels can be falsely lowered by laboratory artifacts met in conditions such as hyperlipidemia and elevated serum protein levels (intravenous immunoglobulin administration, monoclonal gammopathies), where the excessive lipids/proteins dilute the aqueous phase of the extracellular compartment [41]. Hypertonic hyponatremia (plasma osmolality > 295 mOsm/L) is caused by an increased osmotic pressure in the extracellular compartment which is commonly seen in the case of hyperglycemia and the administration of radiocontrast media, mannitol, ethanol, methanol. For every 100 mg/dl rise in serum glucose level above a standard serum glucose concentration of 100 mg/dl, there is a serum sodium level decrease by 2.4 mEq/L [42]. Before taking any action for the correction of hyponatremia, it is necessary to identify and manage these underlying causes.

**Figure 3:**
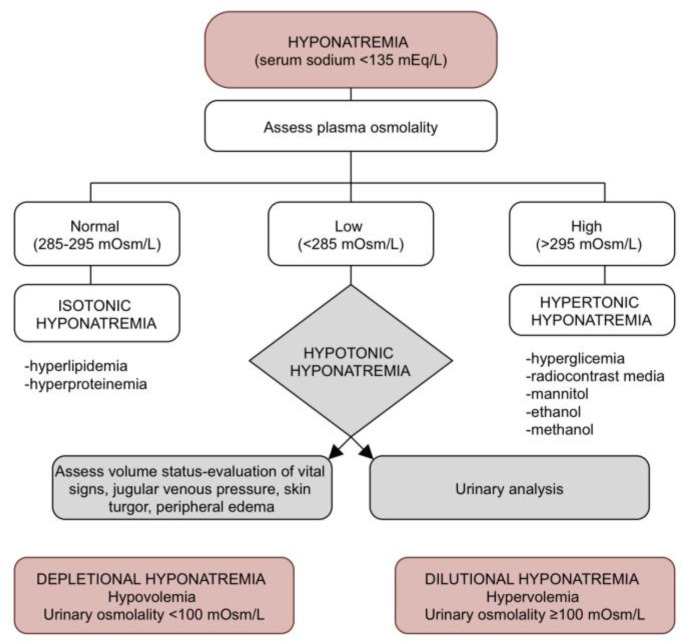
Diagnosis algorithm in heart failure associated hyponatremia.

Once hypotonic hyponatremia is identified (plasma osmolality < 285 mOsm/L), the next step is to differentiate between dilutional and depletional hyponatremia, taking into consideration the differences in therapy approach. This can be done by knowing the medical history of the patient (use of diuretics, salt-restricted diets), by assessing the volume stats of the patient (clinical signs of hypovolemia or hypervolemia), and by measuring urine osmolality [43]. The diagnosis algorithm in heart failure associated hyponatremia is provided below (Figure 3).

## 6. Treatment of Acute and Severe Hyponatremia 

Severe hyponatremia is defined by serum sodium levels <125 mEq/L [24]. Acute hyponatremia (which has an onset <48 h) is often accompanied by severe neurological symptoms, due to the incapability of the adaptive mechanisms to counteract the brain edema [7]. The management of patients with severe symptoms, including vomiting, seizures, confusion, and coma must be performed in an environment where close biochemical and clinical monitoring can be provided, and hyponatremia must be treated urgently regardless of the acute or chronic onset [44].

The European Society of Endocrinology guidelines for the treatment of severe hyponatremia recommend for the first-hour management a 5 mmol/L increase in serum sodium levels, by “prompt i.v. infusion of 150 mL 3% hypertonic saline over 20 min” (p. 28) [40] with checking the serum sodium levels after 20 min. In case of an adequate increase of 5 mmol/L in serum sodium concentration and improvement of symptoms in the first hour, infusion of the hypertonic saline should be stopped and serum sodium levels increase should not exceed “10 mmol/L during the first 24 h and an additional 8 mmol/L every 24 h thereafter until the serum sodium concentration reaches 130 mmol/L“ (p. 28) [40]. Close monitoring of serum sodium levels is needed (after 6 and 12 h and daily afterwards) in order to avoid overcorrection [40,45]. If the symptoms still persist after a proper correction of serum sodium levels in the first hour, the 3% hypertonic saline infusion should be continued and serum sodium level concentration should be checked every 4 h. The goal is to obtain an additional increase of 1 mmol/L/hour [40].

If hypokalemia and hypomagnesemia are present, they also need to be corrected [43]. In addition to the higher risk of arrhythmic death associated with potassium depletion, low potassium serum levels promote the development of hyponatremia by shifting sodium into the cell. Magnesium is involved in the normal function of Na^+^/K^+^-ATPase which pumps Na^+^ out of the cells, so a depletion in magnesium level can also contribute to lower extracellular sodium levels [46]. 

## 7. Treatment of Dilutional Hyponatremia

Heart failure remains a major cause of mortality around the world, despite the significant advances in therapies. [47] The main goal in the acute treatment of dilutional hyponatremia is to promote free water excretion in order to achieve normal serum sodium levels. This can be done by increasing distal nephron flow or by antagonizing AVP effects.

### 7.1. Loop Diuretics

The first-line treatment of acute decompensated heart failure with dilutional hyponatremia and congestion is represented by loop diuretics, as they increase the distal nephron flow and reduce tonicity in the renal interstitium, promoting free water excretion. Treatment must be started with intravenous diuretics in order to obtain a fast response, and once the patient is stabilized, transition to oral treatment should be carried out [48]. Premature discharge of a patient with acute decompensated heart failure can have severe consequences [49]. The initial dose of intravenous diuretic treatment depends on the presence or absence of diuretic therapy before the decompensation episode. It is necessary to evaluate the diuretic response shortly after, and if there is an unsatisfactory response, the dose can be doubled. If there is still an insufficient diuretic response, a combination of diuretic therapies may be considered [48]. In this case, acetazolamide is a good option for combinational diuretic treatment, taking into consideration the strong aquaretic effect of this diuretic associated with the property of mobilization of Na^+^ from the interstitial space into the vascular space [50].

Despite the increasing dose of diuretics or combined diuretic therapy, some patients with acute decompensated heart failure develop diuretic resistance and a worsening of their renal function [51]. Even though salt restriction is usually recommended in heart failure due to the risk of congestion exacerbation, it can also cause an increased neurohormonal activation by increasing sodium avidity signaling. Miller et al., in a study on a canine model conducted over a period of 38 days, observed that low dietary sodium (0.25 g/day) was associated with increased aldosterone levels that can lead to the development of symptoms in chronic heart failure. This aldosterone activation was also observed in normal and high sodium dietary groups, but it occurred earlier (10 days) in the low dietary sodium group. It was also associated with a higher decrease in the ejection fraction compared with the high dietary sodium group [52]. 

### 7.2. Hypertonic Saline Solution

In recent years, association between loop diuretics and hypertonic saline solutions in the management of heart failure and associated hyponatremia became an area of interest in many studies. Tuttolomondo A. et al., observed that administration of a high dose of furosemide in association with hypertonic saline solution led to a decrease in natriuretic peptide and cytokine plasma levels (TNF-α, IL-1β, IL-6) [53]. Griffin M. et al., made a retrospective analysis in patients receiving 150 mL of 3% NaCl over 30 min, administered simultaneously with high doses of loop diuretics. Vital signs, capillary oxygen saturation, cardiac, renal, and pulmonary functions were monitored in all patients before and after the administration, identifying objective improvements in patient status [54]. In order to summarize the available information regarding the effects of hypertonic saline association to loop diuretics in the management of heart failure, we conducted a literature research in PubMed database, with “hypertonic saline” and “heart failure” as search terms. We selected the studies published in the period of 2000–2022 which included patients with acute decompensated heart failure who had at least one of the following outcomes: serum sodium levels, weight loss, renal function, mortality rate, and hospitalization time (Table 2). The main beneficial effects of association between loop diuretics and hypertonic saline solutions are represented by improvements in serum sodium levels with a low/no risk of overcorrection, increased diuretic efficiency, fluid and weight loss, improvement in renal function, shorter hospitalization, and lower mortality rates [53,54,55,56,57,58,59,60,61,62,63,64,65,66].

### 7.3. Fluid Restriction

**Figure 4:**
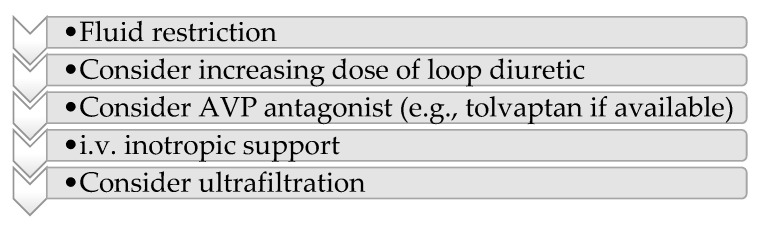
2021 European Society of Cardiology Guidelines for the diagnosis and treatment of acute and chronic failure.

The first recommendation of the European Society of Cardiology 2021 guidelines in managing hypervolemia (Figure 4) in heart failure is represented by fluid restriction (less than 800–1000 mL/day) [48]. 

Normally, by limiting the intake of free water there should be an increase in the serum sodium levels. Dunlap M.E. et al., compared the effects of fluid restriction, no therapy, and specific therapy in hypervolemic hyponatremia. The results suggested that fluid restriction has a small effect on serum sodium levels (only a 2 mEq/L raise in 24 h) compared to specific therapies (as hypertonic saline, tolvaptan) which were associated with >5 mEq/L raise in serum sodium levels in 24 h. Furthermore, fluid restriction in monotherapy led to persistence of hyponatremia at hospital discharge in the majority of patients [67]. Another problem associated with water restriction is that many patients may find difficulties in adapting to a strict regimen, taking into consideration that thirst is a common find in heart failure [68]. There is a need for more data to assess long-term efficacy of fluid restriction and the benefits are clearly enhanced by also using specific therapies. The FRESH-UP study (Fluid REStriction in Heart failure vs liberal fluid Uptake) is a randomized, controlled, open-label, multicenter trial which was started in order to address the gap in evidence regarding the real benefits of fluid restriction in patients with heart failure. The goal of the study is to investigate the impact of fluid restriction of 1500 mL/day versus liberal fluid intake on quality of life (QoL) at 3 months after randomization. Patient recruitment started in May 2021 and is expected to continue until 2023. The results of the FRESH-UP study may lead to a more evidence-based guideline for fluid restriction in chronic heart failure and may impact the quality of life of these patients [69].

### 7.4. Vaptans

For the purpose of this review, a database was created by compiling all articles that referenced the role of vaptans in heart failure. In particular, the review focused on studies where patients with heart failure were administered vaptans and excluded any studies that referenced or tried to replicate the initial paper. The field was narrowed further by only taking into account trials measuring the sodium levels by comparing vaptan treatment with a placebo group or when tolvaptan was compared with furosemide.

As mentioned before, the arginine vasopressin system is the main cause for the occurrence of dilutional hyponatremia in patients with congestive heart failure. This made it necessary to study vasopressin antagonists, called vaptans [43,70]. Vaptans inhibit AVP receptors, causing water excretion without loss of electrolytes, which differentiate them from diuretics. There are V2 receptor-selective antagonists (tolvaptan and lixivaptan) and both V2 and V1a receptor antagonists (conivaptan), available for oral or intravenous route of administration [71]. Of these, tolvaptan and conivaptan have been the most studied.

Tolvaptan has been extensively studied for its beneficial effects in heart failure and other conditions associated with hyponatremia. Many meta-analyses concluded that short- and long-term use of tolvaptan in heart failure was safe while it has beneficial effects such as weight loss, high urine output, symptomatic improvement and restoration of serum sodium levels without altering the kidney function. Several other studies also showed improvement in serum sodium with the use of vaptans, but outcomes in the long-term remained unknown [7]. 

Conivaptan with its hemodynamic effects has been evaluated in small studies with chronic heart failure patients on standard treatment. The addition of conivaptan to furosemide showed an increase in urine volumes and greater weight loss, with a favorable tolerability, without any alteration of mean arterial pressure, heart rate, or serum electrolytes [71]. It is an attractive option for neurological patients unable to receive oral medications and has been found in preliminary data to reduce cerebral edema and intracranial pressure (ICP) via the V1a receptor and the aquaporin channel expressed by astrocytes [72]. Conivaptan is an inhibitor of cytochrome P450 3A4 (CYP3A4), which can interact with many other drugs, so its administration with CYP3A4 inhibitors such as ketoconazole, clarithromycin, and ritonavir is contraindicated. Additionally, its administration can increase plasma concentration of simvastatin, digoxin, midazolam, and amlodipine. Tolvaptan has less potential for drug–drug interaction [73].

The following studies investigated tolvaptan as an alternative therapy to loop diuretics in chronic and acute failure with hyponatremia (Table 3).

The AQUA-AHF trial case (Tolvaptan vs. Furosemide-Based Diuretic Regimens in Patients Hospitalized for Heart Failure with Hyponatremia) compared oral tolvaptan versus intravenous furosemide administration, demonstrating higher values of serum sodium in patients treated with vasopressin inhibitors in the context of similar diuresis to those treated with furosemide [75].

Trial studies such as the Study of Ascending Levels of Tolvaptan, SALT-1 (US) and SALT-2 (international), in which 205 and 243 patients respectively were enrolled, evaluated the safety and efficacy of tolvaptan administration in HF, cirrhosis, and SIADH (syndrome of inappropriate antidiuretic hormone secretion) compared to placebo. The results demonstrated that sodium levels increased in patients with hyponatremia due to depletion after administration of tolvaptan, but no long-term efficacy was shown or differences in mortality of patients treated with AVP antagonists compared to those not treated with it [76]. The EVEREST trial case (Efficacy of Vasopressin Antagonist in Heart Failure Outcome Study with Tolvaptan) follows the acute and chronic effects of fixed-dose tolvaptan 30 mg/day [77]. The TACTICS-HF (The Targeting Acute Congestion with Tolvaptan in Congestive Heart Failure) and ACTIV in CHF (Acute and Chronic Therapeutic Impact of Vasopressin antagonist in Congestive Heart Failure) trial case showed a rapid increase in serum sodium secondary to increase urine output [78,79]. The SECRET of CHF (Study to Evaluate Challenging Responses to Therapy in Congestive Heart Failure) evaluated the short-term clinical effects of tolvaptan in patients hospitalized for acute HF who had challenging volume management [80,81]. Results of the mentioned studies are provided in Table 4. 

In order to avoid an excessive correction of serum sodium concentration in patients who start the treatment with vaptans, serum sodium levels should be closely monitored and fluid intake should not be restricted [82]. They are contraindicated in hypovolemic hyponatremia because of the possibility of precipitating hypotension and renal failure [83]. Vasopressin receptor antagonists are, in general, well tolerated drugs. The most frequent side effects are thirst, pollakiuria, and dry mouth [84].

### 7.5. Other Therapy Options

Inotropes and vasodilatator therapy can be used in order to improve the cardiac output in severe heart failure, by stimulation of cardiac contractility and reduction of the afterload [85]. Pathophysiologically, by improving the renal blood flow, a correction of hyponatremia can be achieved. Mullens W. E. et al., demonstrated that in patients with low-output heart failure, vasodilator therapy associated with optimal medical therapy during hospitalization led to a greater improvement in hemodynamic measurements during hospitalization and lower rates of all-cause mortality. By using sodium nitroprusside in patients with ADHF and low-output states admitted for intensive medical therapy including vasoactive drugs, they obtained higher mean central venous pressure and pulmonary capillary wedge pressure, without a worsening in the renal function during hospitalization [86]. Studies have also shown that the addition of oral vasodilators (hydralazine, nitrates) to standard neurohormonal blockade in discharged patients with adequate systemic blood pressures is associated with lower all-cause mortality with intermediate and long-term benefits [87].

Angiotensin-converting enzyme inhibitors (ACEI) represent one of the pharmacological treatments indicated in patients with heart failure with reduced ejection fraction, as they play an important role in reducing the risk of hospitalization, in improving symptoms and exercise capacity and in increasing survival. They should be used in all patients unless not tolerated or contraindicated [7]. It is suggested that ACEI are effective in hypotonic dilutional hyponatremia in heart failure, because of their ability to increase the renal blood flow and to decrease proximal tubular sodium reabsorption. Small studies showed that mild hyponatremia does not represent a contraindication for the use of ACEI [88] and up-titration should be promoted as long as the renal function is monitored and within normal parameters.

### 7.6. Ultrafiltration

Extracorporeal ultrafiltration (UF) is an emerging alternative therapy for treating patients with acute decompensated heart failure who fail to respond to diuretic-based strategies [48]. UF equipment is similar to that for hemodialysis. It allows excess fluid removal through a semipermeable membrane [89]. There are multiple ultrafiltration approaches, with the veno–venous ultrafiltration technique being the preferred one [90]. Compared to diuretic therapy, UF is associated with a limited/no neurohormonal activation and it is able to restore the diuretic responsiveness [89]. Another benefit of UF is that it allows the production of an isotonic ultrafiltrate, with no contribution to electrolyte abnormalities [91]. In recent years, multiple random clinical trials, such as AVOID-HF (Aquapheresis versus Intravenous Diuretics and Hospitalization for Heart Failure), CUORE (Continuous Ultrafiltration for Congestive Heart Failure), CARESS-HF (Cardiorenal Rescue Study in Acute Decompensated Heart Failure), ULTRADISCO (ULTRAfiltration vs. DIureticS on clinical, biohumoral and haemodynamic variables in patients with deCOmpensated heart failure), UNLOAD (Ultrafiltration versus Intravenous Diuretics for Patients Hospitalized for Acute Decompensated Congestive Heart Failure), RAPID-CHF (Relief for Acutely Fluid-Overloaded Patients with Decompensated Congestive Heart Failure), investigated the effects of UF on renal function, congestion, hospitalization length, readmission, and mortality rates, compared with diuretic therapy in patients with heart failure [92,93,94,95,96,97]. Even though they identified the potential beneficial effects of UF, due to the heterogeneity of protocols and study results, there is still a need for future studies to fill the gaps in knowledge. Moreover, taking into consideration that there are no precise guidelines and algorithms for the selection of patients that may undergo ultrafiltration, this procedure still needs to be carefully examined.

## 8. Conclusions

Hyponatremia is a major clinical concern during and following hospitalizations of patients with heart failure, being associated with poor short- and long-term outcomes. An acute decrease in serum sodium levels can have fatal consequences for the patient, while chronic hyponatremia leads to impairments that affect the quality of life.

Confirmation of plasma hypotonicity and differentiation between dilutional and depletional hyponatremia are the first steps for the correct management of the patient with heart failure and hyponatremia. Depletional hyponatremia is managed with saline administration The main treatment options for dilutional hyponatremia are represented by fluid restriction and the use of hypertonic saline with loop diuretics. Prompt administration of hypertonic saline in both acute and severe hyponatremia may save lives. The compliance in fluid restriction can be reduced by the increased thirst which occurs in patients with heart failure. Moreover, studies suggested that fluid restriction has a small effect on serum sodium levels.

There is clearly a need for more potentially promising treatment options for this condition. The vaptan class is an interesting option, mainly because of the significant free water loss that can be achieved. In recent years, many studies investigated the utility of vasopressin receptor antagonists for refractory hypervolemic hyponatremia. Even tough beneficial short-term effects were identified, future studies are required to assess the impact of vaptans on long-term outcomes.

## Figures and Tables

**Table 1:** Signs and symptoms of osmotic demyelination syndrome.

Signs and symptoms of ODS	- attention deficit - memory loss - dysarthria - dystonia - ataxia - mutism	- parkinsonism - catatonia - tremor - ‘locked-in syndrome’ - seizures - coma

**Table 2:** Studies on hypertonic saline in patients with acute decompensated heart failure.

First Author (Ref. #)	Year	Type of Study	Treatment	Effects of Hypertonic Saline Association to Loop Diuretics
**Tuttolomondo et al.** **[53]**	2021	Randomized Controlled Trial 141 patients	30 min of i.v. infusion of furosemide (120–250 mg) + HSS (150 mL of 1.4–4.6% NaCl) twice a day for 6 days versus 30 min of i.v. infusion of furosemide (120–150 mg) twice a day without HSS for 6 days.	Increase in diuresis, weight loss; reduction in the serum markers of atrial stretching, fibrosis and inflammation.
**Griffin et al.** **[54]**	2020	Retrospective Study 40 patients	i.v. 150 mL of 3% NaCl over 30 min + high doses of loop diuretics.	Improvements in urine output, weight loss, diuretic efficiency, renal function; increase in serum sodium levels; no discernible deterioration in respiratory status or overcorrection of hyponatremia.
**Wan et al.** **[55]**	2017	Randomized Controlled Trial 264 patients	i.v. 1-h infusion of furosemide (100 mg) plus HSS (100 mL) twice a day and severe water restriction (< 500 mL) vs. i.v. furosemide (100 mg) twice a day and severe water restriction (< 500 mL) without HSS.	Increase in urination, reduction of hospitalization time and costs; higher average readmission time; lower mortality rate.
**Lafrenière et al.** **[56]**	2017	Prospective Study 47 patients	i.v. infusion of 250 mg furosemide plus 150 mL HSS 3% NaCl twice a day for a mean duration of 2.3 days.	Greater weight loss per day of treatment.
**Yayla et al.** **[57]**	2015	Randomized Controlled Trial 43 patients	Continuous infusion of 160 mg furosemide for 16 h/day versus bolus injections of 80 mg furosemide twice a day versus administration of 160 mg furosemide plus HSS as an infusion for 30 min once a day.	Significantly shorter hospitalization.
**Paterna et al.** **[58]**	2015	Randomized Controlled Trial 40 patients	i.v. furosemide (125 mg/250 mg/500 mg) diluted in 150 mL of normal saline. (0.9%) versus the same furosemide dose diluted in 150 mL of HSS (1.4%).	Increased total urine output, sodium excretion, urinary osmolality, and furosemide urine delivery.
**Okuhara et al.** **[59]**	2014	Randomized Controlled Trial 44 patients	1.7% HSS with 40 mg furosemide versus glucose 5% with 40 mg furosemide.	Favorable diuresis through increasing glomerular filtration rate.
**Issa et al.** **[60]**	2013	Randomized Controlled Trial 34 patients	Three-day course of 100 mL HSS (NaCl 7.5%) twice a day versus placebo.	Improvement in glomerular and tubular defects; attenuation of heart failure-induced kidney disfunction.
**Parrinello et al. ** **[61]**	2012	Randomized Controlled Trial 248 patients	i.v. 30 min infusion of 250 mg furosemide twice a day with versus without HSS (1.4–4.6% NaCl).	Significant improvement in renal function, hydration state, pulmonary capillary wedge pressure, end diastolic volume, ejection fraction; increase in serum sodium levels; significant reduction in body weight, cardiac troponin I and brain natriuretic peptide, and hospitalization time.
**Parrinello et al.** **[62]**	2011	Randomized Controlled Trial 133 patients	i.v. infusion of 250 mg furosemide plus 150 mL 3% hypertonic saline twice a day and light sodium restriction (120 mmol) versus i.v. infusion of 250 mg furosemide twice a day and low sodium diet (80 mmol).	Significant improvement in renal function; increase in diuresis, natriuresis and serum sodium levels; faster reduction of echocardiographic pulmonary capillary wedge pressure.
**Paterna et al.** **[63]**	2011	Randomized Controlled Trial 1771 patients	i.v. infusion of 250 mg furosemide plus 150 mL 3% hypertonic saline twice a day and light sodium restriction (120 mmol) versus i.v. infusion of 250 mg furosemide twice a day and low sodium diet (80 mmol).	Increase in diuresis, natriuresis and serum sodium levels; reduction of hospitalization time; lower rate of readmission; lower rate of mortality.
**Paterna et al.** **[64]**	2005	Randomized Controlled Trial 94 patients	i.v. 500–1000 mg furosemide with 150 mL HSS (1.4–4.6% NaCl) twice a day versus i.v. 500-1000 mg furosemide twice a day without HSS.	Increase in diuresis, natriuresis and serum sodium levels; decrease in brain natriuretic peptide levels; reduction in hospitalization time and readmission rate.
**Licata et al.** **[65]**	2003	Randomized Controlled Trial 107 patients	i.v. 30 min infusion of 500–1000 mg furosemide with 150 mL HSS (1.4–4.6% NaCl) twice a day versus i.v. 500–1000 mg furosemide twice a day without HSS.	Significant increase in diuresis and natriuresis; increase in serum sodium levels; mortality reduction.
**Paterna et al.** **[66]**	2000	Randomized Controlled Trial 60 patients	i.v. 500–1000 mg furosemide with 150 mL HSS (1.4–4.6% NaCl) twice a day versus i.v. 500–1000 mg furosemide twice a day without HSS.	Increase in diuresis, natriuresis, and serum sodium levels; decrease in serum creatinine; reduction of hospitalization time; maintaining the obtained results over time.

HSS = hypertonic saline solution; i.v. = intravenous.

**Table 3:** Tolvaptan as an alternative therapy to loop diuretics in chronic and acute failure with hyponatremia [74,75].

	Udelson 2011 [74]	AQUA-AHF 2020 [75]
**Enrolled patients**	83 Multicenter, randomized, double-blind placebo-controlled.	33 Prospective, randomized, open-label, parallel-group, single center study.
**Inclusion** **criteria**	NYHA class II or III HF, systolic dysfunction (EF ≤ 40%) and signs of congestion (edema, rales).	Acute congestive HF and a serum Na < 135 mmol/L.
**Design/tolvaptan** **dosing**	4 groups: tolvaptan 30 mg, furosemide 80 mg, a combination of tolvaptan 30 mg/furosemide 80 mg and placebo for 7 days (+ standard therapy).	Randomized to receive tolvaptan 30 mg orally daily or furosemide 5 mg/h intravenously for 24 h, after which treatment could be escalated.
**Primary end point results**	Reduction of body weight was similar in all groups.	No significant differences in median urine output or net fluid balance between groups at 24 h.
**Secondary end point results**	An increase in serum sodium within the normal range was also observed in tolvaptan-treated group when compared with placebo or furosemide group.	Oral tolvaptan was associated with similar, but not superior diuresis compared with intravenous furosemide for acute HF with concomitant hyponatremia. In contrast to conventional diuretics, exacerbation of hyponatremia is unlikely with tolvaptan.

**Table 4:** Studies of tolvaptan versus placebo in heart failure and sodium evaluation endpoint.

	EVEREST 2007	TACTICS 2017	SECRET of CHF 2017	ACTIV in CHF 2004	SALT 1 and SALT 2 2006
**Enrolled** **patients**	4133 Phase III clinical trial	257 Multicenter, randomized double-blind, placebo controlled trial	250 Randomized, double-blind, placebo-controlled trial	319 Prospective, international, multicenter, randomized controlled trial	448 Multicenter, randomized, double-blind, placebo controlled trial
**Enrollment time after admission**	Within 48 h	Within 24 h	Within 36 h	Within 72 h	
**Inclusion criteria**	NYHA class III or IV ≥ 2 signs/symptoms of fluid overload (dyspnea, peripheral edema, and JVD) EF ≤ 40%.	Dyspnea at rest, BNP > 400 pg/mL, NT-proBNP > 2000 pg/mL, orthopnea or peripheral edema or JVD or pulmonary rales or congestion on chest X-ray, Serum sodium ≤ 140 mmol/L.	NYHA class III or IV or acute decompensated HF (dyspnea and either impaired renal function, diuretic resistance or hyponatremia).	Decompensated HF, EF < 40%, fluid overload requiring hospitalization.	Hyponatremia arising from CHF, cirrhosis and SIADH.
**Exclusion criteria**	SBP < 90 mmHg, Serum creatinine > 3.5 mg/dL or dialysis, Serum potassium > 5.5 mEq/L.	Serum sodium > 140 mEq/L, SBP < 90 mmHg, Serum creatinine> 3.5 mg/dL or currently undergoing renal replacement therapy.	SBP < 90 mmHg, Serum sodium > 144 mEq/L, Serum creatinine > 3.5 mg/dL or dialysis.	Recent myocardial infarction, recent cardiac surgery, SBP < 110 mmHg, life-threatening arrhythmias, severe renal impairment.	
**Tolvaptan dosing**	30 mg of tolvaptan in addition of standard therapy for a minimum of 60 days.	Tolvaptan 30 mg orally vs. placebo given at 0, 24, and 48 h (e.g., 3 doses) in patients hospitalized for AHF and congestion in addition to a fixed dose of furosemide.	30 mg of tolvaptan vs placebo during hospitalization (for up to 7 days).	Tolvaptan orally (30, 60 or 90 mg/day) or placebo (randomized 1:1:1:1) + standard therapy, including diuretics. Study drug continued for 60 days.	Tolvaptan 15 mg with titration to 30 and 60 mg as needed to correct sodium over 30 days, with a follow-up visit 7 days after study end.
**Primary end point results**	Greater reduction in body weight at day 1 and day 7 (or at time of discharge).	No difference in the percentage of patients who showed moderate or marked clinical improvement with tolvaptan (without need for rescue therapy).	No difference in self –assessment of dyspnea at 8 and 16 h.	Greater urine output and weight loss at 24 h after initial dose of tolvaptan when compared to the placebo group.	Serum sodium concentration increased more in tolvaptan group during the first 4 days and after the full 30 days of therapy. Within 7 days after stopping tolvaptan, serum sodium returned to the placebo levels.
**Secondary end point results**	Improved dyspnea and peripheral edema. No excess of renal failure or hypotension in the tolvaptan group. Tolvaptan significantly increased serum sodium levels at day 7.	More weight loss and urine output at 24 h with tolvaptan. No difference in dyspnea relief. Serum sodium increased by an average of 3 mmol/L in the tolvaptan group.	Greater weight reduction with tolvaptan. By day 3 there was a greater reduction in dyspnea in the tolvaptan group. Hyponatremia as an adverse event in tolvaptan group was reported, but without statistical significance compared to placebo.	Less dyspnea at the time of discharge. No difference in progression of HF. Tolvaptan use reduced the amount of loop diuretic used. No difference in HR, BP, or renal function. Tolvaptan was associated with normalization of serum sodium in patients with hyponatremia.	

NYHA = New York Heart Association; JVD = jugular vein distension; BNP = brain natriuretic peptide; NT-proBNP = N-terminal prohormone of brain natriuretic peptide; CHF = chronic heart failure; SIADH = the syndrome of inappropriate antidiuretic hormone secretion; SBP = systolic blood pressure, AHF = acute heart failure, HR = heart rate; BP = blood pressure.

## Data Availability

Not applicable.
